# Ethnicity data resource in population-wide health records: completeness, coverage and granularity of diversity

**DOI:** 10.1038/s41597-024-02958-1

**Published:** 2024-02-22

**Authors:** Marta Pineda-Moncusí, Freya Allery, Antonella Delmestri, Thomas Bolton, John Nolan, Johan H. Thygesen, Alex Handy, Amitava Banerjee, Spiros Denaxas, Christopher Tomlinson, Alastair K. Denniston, Cathie Sudlow, Ashley Akbari, Angela Wood, Gary S. Collins, Irene Petersen, Laura C. Coates, Kamlesh Khunti, Daniel Prieto-sAlhambra, Sara Khalid, on behalf of the CVD-COVID-UK/COVID-IMPACT Consortium

**Affiliations:** 1 Centre for Statistics in Medicine, Botnar Research Centre, Nuffield Department of Orthopaedics, Rheumatology and Musculoskeletal Sciences (NDORMS), University of Oxford, Oxford, UK; 2 Institute of Health Informatics, 222 Euston Road, London, NW1 2DA, University College London, London, UK; 3 British Heart Foundation Data Science Centre, Health Data Research UK, London, UK; 4 University College London Hospitals Biomedical Research Centre, University College London, London, UK; 5 UK Research and Innovation Centre for Doctoral Training in AI-enabled Healthcare Systems, University College London, London, UK; 6 University Hospitals Birmingham NHS Foundation Trust (NHSFT), Birmingham, UK; 7 Population Data Science, Swansea University Medical School, Faculty of Medicine, Health & Life Science, Swansea University, Swansea, Wales UK; 8 British Heart Foundation Cardiovascular Epidemiology Unit, Department of Public Health and Primary Care, University of Cambridge, Cambridge, UK; 9 Victor Phillip Dahdaleh Heart and Lung Research Institute, University of Cambridge, Cambridge, UK; 10 Department of Primary Care and Population Health, UCL, London, NW3 2PF UK; 11 Department of Clinical Epidemiology, Aarhus University, Aarhus N, Aarhus, 8200 Denmark; 12 Nuffield Department of Orthopaedics, Rheumatology and Musculoskeletal Sciences (NDORMS), University of Oxford, Oxford, UK; 13 Diabetes Research Centre, University of Leicester, Leicester, UK; 14 Department of Medical Informatics, Erasmus MC University Medical Centre Rotterdam, Rotterdam, The Netherlands

**Keywords:** Epidemiology, Epidemiology

## Abstract

Intersectional social determinants including ethnicity are vital in health research. We curated a population-wide data resource of self-identified ethnicity data from over 60 million individuals in England primary care, linking it to hospital records. We assessed ethnicity data in terms of completeness, consistency, and granularity and found one in ten individuals do not have ethnicity information recorded in primary care. By linking to hospital records, ethnicity data were completed for 94% of individuals. By reconciling SNOMED-CT concepts and census-level categories into a consistent hierarchy, we organised more than 250 ethnicity sub-groups including and beyond “White”, “Black”, “Asian”, “Mixed” and “Other, and found them to be distributed in proportions similar to the general population. This large observational dataset presents an algorithmic hierarchy to represent self-identified ethnicity data collected across heterogeneous healthcare settings. Accurate and easily accessible ethnicity data can lead to a better understanding of population diversity, which is important to address disparities and influence policy recommendations that can translate into better, fairer health for all.

## Introduction

Health inequity is described by disparities in health status between individuals, such as prevalence of comorbidities, life expectancy, access to and quality of care services and treatments, and risk behaviours such as smoking and alcohol consumption. These factors can be influenced by age, sex, ethnicity, disability, socio-economic status, geographical location, and education, among others^1^. For example, many of these determinants were risk factors for infection severity, complications, and mortality during the COVID-19 pandemic^2–4^.

“Ethnicity” commonly refers to terms used to self-report an individual’s own perceived ethnic group and cultural background. This multidimensional, evolving concept can comprise physical appearance, race, culture, language, religion, nationality and identity elements, and is not always captured in electronic health records. Additionally, when recorded, ethnicity is often inaccurately coded, especially for groups other than the predominant group(s) in a given population^5,6^. Ethnicity classifications also change over time, limiting comparability with population-level census data^7^. In UK health records, although there are hundreds of heterogenous ethnicity groups defined in the form of SNOMED-CT codes or Read codes, among others, they are commonly collapsed into five or six categories, in part due to power considerations where fine-grained or granular categories would have smaller sample sizes^8–11^. However, these larger groups may not be equivalent or translate across the world due to differences in population demographics^12,13^. Nonetheless, this oversimplification of categories can result in loss of diversity and precision in studies using ethnicity. Incorrect or unrepresentative ethnicity records risk introducing bias in insights drawn from health data and ensuing literature, ultimately contributing to inappropriate healthcare. Use of population-wide routinely-collected data offers an opportunity to study diverse ethnicity groups in detail with sufficient power, enabling health research to become more inclusive^4,10,11^.

Health inequality was highlighted as a significant issue during the COVID-19 pandemic when individuals from ethnically diverse backgrounds in otherwise predominantly White populations were disproportionately affected by SARS-CoV-2^11^. However, this is an ongoing and multifaceted challenge; one underlying source is bias in health data and ensuing technologies. Understanding and addressing biases in health data is a fundamental first step in addressing this challenge. To improve the understanding of how ethnicity is recorded, mapped, and used in the UK, we explored ethnicity records for completeness, consistency, and granularity in National Health Service (NHS) England’s Secure Data Environment (SDE) service for England (UK) accessed via the BHF Data Science Centre’s CVD-COVID-UK/COVID-IMPACT Consortium^14^.

## Methods

### Data sources and linkages

NHS England maintains an SDE for secure access to anonymised patient-level electronic health records for England with linkages to primary-, secondary-, and tertiary-care data sources for research purposes^15,16^. NHS England’s Master Person Service facilitates the linkage between the SDE data sources through the NHS number (a unique 10 digit healthcare identifier), date of birth, and sex^15,17^.

This study focused on the General Practice Extraction Service (GPES) Data for Pandemic Planning and Research (GDPPR) data sources, a primary-care dataset for England that collects information from all individuals who are currently registered with a general practitioner (GP) practice and any individual who died on or after 1^st^ November 2019. GDPPR does not include individuals who died before November 2019 for ethical reasons as those individuals are considered out of scope for COVID-19 research. It is accessible through the NHS England SDE^18^ (formerly NHS Digital TRE^15,16^). Data include diagnoses, prescriptions, treatments, outcomes, vaccinations, and immunisations^19,20^. GDPPR covers 98% of English GP practices across all relevant GP computer system suppliers (TPP, EMIS, Cegedim (formerly called Vision or In Practice Systems), and Microtest)^15^.

### Data sets used

To evaluate and curate the ethnicity data available in the NHS England SDE^15,16^, we used the following three linked datasets:

- Primary care data: the General Practice Extraction Service (GPES) Data for Pandemic Planning and Research (GDPPR)^19,20^.
- Hospital admissions data: Hospital Episode Statistics for admitted patient care (HES-APC)
- Mortality information from the Office for National Statistics (ONS): Civil Registration of Deaths.

GDPPR was used to select the individuals included in the study. It was the main source to obtain ethnicity data, and all variables included in the study except death, which was obtained from the Civil Registration of Deaths. HES-APC was used as a second source to obtain ethnicity data.

### Data access

A data sharing agreement issued by NHS England for the CVD-COVID-UK/COVID-IMPACT research programme (ref: DARS-NIC-381078-Y9C5K) enables accredited, approved researchers from institutions party to the agreement to access data held within the NHS England SDE service for England.

### Source codes for ethnicity

**Fig. 1:**
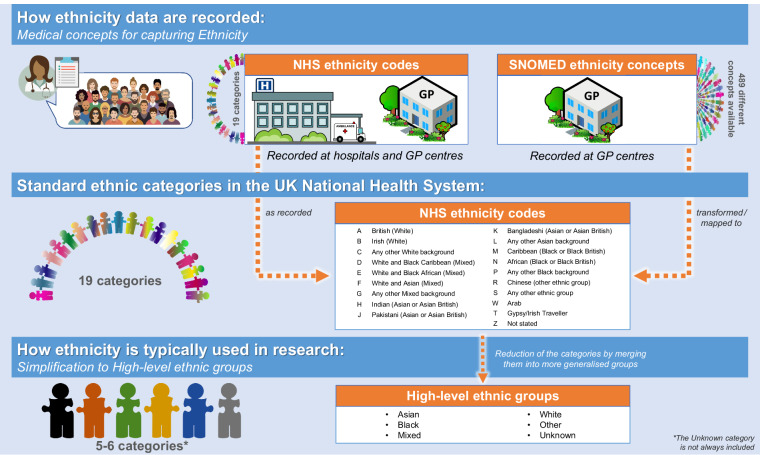
How ethnicity is collected in the UK and typically used for research. The A-Z letters are the nomenclature observed in the data to represent the NHS ethnicity codes. Abbreviations: High-level ethnicity groups, general ethnicity classification groups from the Office for National Statistics commonly used in research; NHS, National Health Service in the UK; SNOMED, SNOMED-CT records containing ethnicity concepts.

Ethnicity is recorded in health records using the following medical concepts (Fig. 1):

- SNOMED concepts: The Systematized Nomenclature of Medicine Clinical Terms is a standardised vocabulary for the recording of patients’ clinical information in electronic health records. It is used across NHS practices and healthcare providers^21^. We focused on GDPPR records containing SNOMED-CT^22^ UK Edition ethnicity concepts. Any mention of SNOMED concepts in this paper directly refers to these codes.
- NHS ethnicity codes: Standard ethnicity categories defined in the NHS England Data Dictionary^23^, using A-Z notation. Ethnicity fields in the NHS tables may use different census classifications, therefore NHS ethnicity code notation may differ slightly depending on which census it is based on. Table 1 summarises the NHS ethnicity codes available in GDPPR, the corresponding categories in HES-APC, and the 2011 and 2021 UK ONS census categories. Mapping of SNOMED concepts to NHS ethnicity codes was provided by NHS England (Table S1).

**Table 1:** NHS ethnicity codes for Ethnicity (A-Z) available in GDPPR and corresponding categories in Census (2021), Census (2011), Hospital Episode Statistics, and notation before 2001.

Census (2021)	Census (2011)	GDPPR (2011)		HES-APC (2001)		Codes from 1995–1996 to 2000–2001	
**White: English, Welsh, Scottish, Northern Irish or British**	White: English/Welsh/Scottish/Northern Irish/British	A	British	A	British (White)	0	White
**White: Irish**	White: Irish	B	Irish	B	Irish (White)	0	White
**White: Gypsy or Irish Traveller**	White: Gypsy or Irish Traveller	T	Traveller	—	—	—	—
**White: Roma**	—	—	—	—	—	—	—
**White: Any other White background**	White: Other White	C	Any other White background	C	Any other White background	0	White
**Mixed or multiple ethnic groups: White and Black Caribbean**	Mixed/multiple ethnic groups: White and Black Caribbean	D	White and Black Caribbean	D	White and Black Caribbean (Mixed)	—	—
**Mixed or multiple ethnic groups: White and Black African**	Mixed/multiple ethnic groups: White and Black African	E	White and Black African	E	White and Black African (Mixed)	—	—
**Mixed or multiple ethnic groups: White and Asian**	Mixed/multiple ethnic groups: White and Asian	F	White and Asian	F	White and Asian (Mixed)	—	—
**Mixed or multiple ethnic groups: Any other Mixed or multiple ethnic background**	Mixed/multiple ethnic groups: Other Mixed	G	Any other Mixed background	G	Any other Mixed background	—	—
**Asian or Asian British: Indian**	Asian/Asian British: Indian	H	Indian	H	Indian (Asian or Asian British)	4	Indian
**Asian or Asian British: Pakistani**	Asian/Asian British: Pakistani	J	Pakistani	J	Pakistani (Asian or Asian British)	5	Pakistani
**Asian or Asian British: Bangladeshi**	Asian/Asian British: Bangladeshi	K	Bangladeshi	K	Bangladeshi (Asian or Asian British)	6	Bangladeshi
**Asian or Asian British: Chinese**	Asian/Asian British: Chinese	R	Chinese	R	Chinese (Other ethnic group)	7	Chinese
**Asian or Asian British: Any other Mixed or multiple ethnic background**	Asian/Asian British: Other Asian	L	Any other Asian background	L	Any other Asian background	—	—
**Black, Black British, Caribbean or African: African**	Black/African/Caribbean/Black British: African	N	African	N	African (Black or Black British)	2	Black - African
**Black, Black British, Caribbean or African: Caribbean**	Black/African/Caribbean/Black British: Caribbean	M	Caribbean	M	Caribbean (Black or Black British)	1	Black – Caribbean
**Black, Black British, Caribbean or African: Any other Black, Black British, or Caribbean background**	Black/African/Caribbean/Black British: Other Black	P	Any other Black background	P	Any other Black background	3	Black - Other
**Other ethnic group: Arab**	Other ethnic group: Arab	W	Arab	—	—	—	—
**Other ethnic group: Any other ethnic group**	Other ethnic group: Any other ethnic group	S	Any other ethnic group	S	Any other ethnic group	8	Any other ethnic group
—	—	Z	Not stated	Z	Not stated	9	Not given
—	—	X	Not known (prior 2013)	—	—	—	—
—	—	99	Not known (2013 onwards)	99	Not known	99	Not known

The Hospital Episode Statistics for admitted patient care (HES-APC) data uses census notation based on the 2001 Census. ‘Z: not stated’ indicates that the person was asked and either refused to provide this information or was genuinely unable to choose a response. ‘X: Not known’ indicates that the person was not asked or was not in a condition to be asked (e.g., unconscious). Abbreviations: NHS, National Health Statistics.

An individual’s ethnicity may be recorded using either SNOMED-CT concepts or NHS ethnicity codes in GDPPR (primary care records), whereas it may only be recorded using the latter in HES-APC (hospital records).

### Other ethnicity classifications

High-level ethnicity groups: Asian/Asian British, Black/African/Caribbean/Black British, Mixed, Other Ethnic Groups, Unknown, and White. Based on ONS ethnicity group high-level category descriptions^13,24^.

**Fig. 2:**
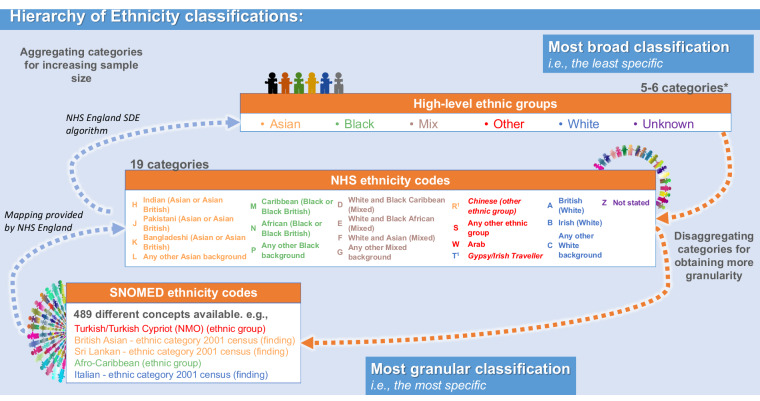
Visual representation of the hierarchy between the three ethnicity classifications, from the broader to the most specific: High-level ethnicity groups, NHS ethnicity codes and SNOMED concepts. The A-Z letters are the nomenclature observed in the data to represent the NHS ethnicity codes. The colours displayed from the High-level ethnicity groups show how the NHS ethnicity concepts and SNOMED-CT can be aggregated into this 6-category classification. The^1^ highlights the different colour for the letters C and T, in respect to the colours of their concepts, Chinese and Gypsy/Irish Traveller, respectively. The colours from the concepts represent the current aggregation algorithm available in the NHS England SDE, whilst the colour of the letters show the aggregation suggested by the UK Office of National Statistics. Abbreviations: *, the Unknown category is not always included; NHS, National Health Service in the UK; SNOMED, SNOMED-CT records containing ethnicity codes; SDE, Secure Data Environment.

The algorithm used within the SDE to condense NHS ethnicity codes and SNOMED concepts to these classifications is provided in Table S2. Figure 2 shows a representation of the hierarchy between the three classification systems, and points where the aggregation is performed by the mapping provided by NHS England or the algorithm used in the SDE.

### Settings and participants

We studied all individuals with a unique patient pseudoidentifier in GDPPR^19,20^ from 1^st^ Jan 1997 until 23^rd^ April 2022. Individuals with an invalid age (i.e., age <0 or ≥115 years old) or missing sex were excluded.

### Covariates

Death date was obtained through civil registration death table which is curated by the ONS and records primary and secondary causes of death using ICD-10. All additional characteristics of individuals were extracted from GDPPR data, which included: age at date of death or age on 23rd April 2022 (date of data extraction), sex, most recent record of residence (i.e., geographical region) in England, body mass index (BMI), index of multiple deprivation (IMD), current smoking status, current alcohol use status, and the presence of any clinical record of atrial fibrillation, acute myocardial infarction, chronic kidney disease, chronic obstructive pulmonary disease (COPD), heart failure, pulmonary embolism, cancer, dementia, diabetes, hypertension, liver disease, obesity, or stroke diagnosis. Geographical region is reported using England’s nine official regions: London, North East, North West, Yorkshire, East Midlands, West Midlands, South East, East, and South West; which were mapped from the Lower Layer Super Output Areas (LSOA).

### Statistical analysis

#### Completeness: missing ethnicity data

**Fig. 3:**
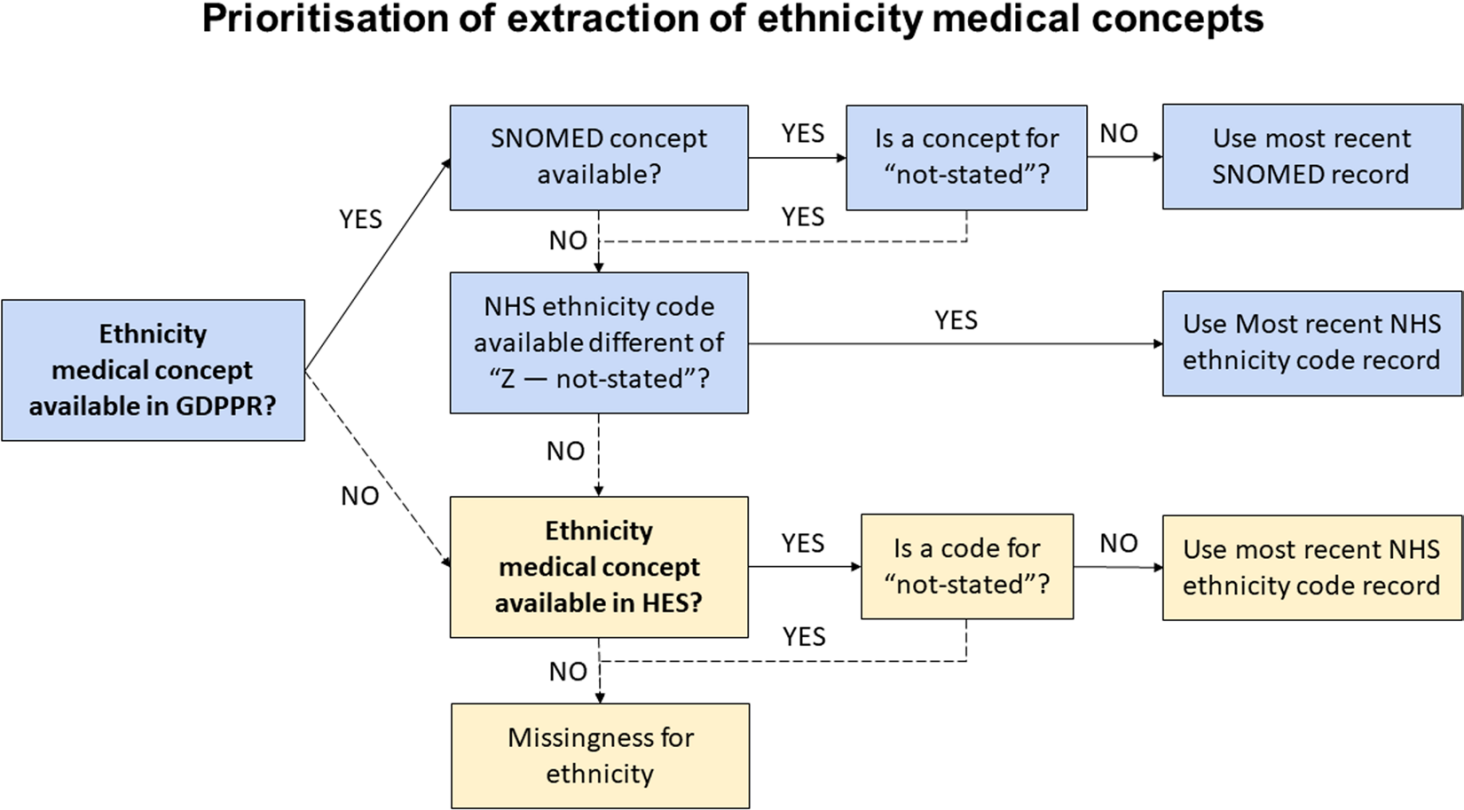
Decision tree of preferred source of ethnicity. Solid arrows mark the preferred option whilst dashed arrows indicate the alternative route. Abbreviations: GDPPR, General Practice Extraction Service (GPES) Data for Pandemic Planning and Research; HES-APC, hospital episode statistics; SNOMED, SNOMED-CT records containing ethnicity codes.

To study completeness, individual-level ethnicity data were extracted from GDPPR using SNOMED concepts and/or NHS ethnicity codes, prioritising SNOMED concepts when available. For individuals with missing ethnicity data in GDPPR, we extracted HES-APC-linked ethnicity data using NHS ethnicity codes (Fig. 3).

Ethnicity missingness was defined as (i) no record in either GDPPR or HES-APC or (ii) an ethnicity code of “not stated” referring to individuals who were asked but preferred not to state their ethnicity and individuals who may not know what to answer. We compared the clinical characteristics of individuals in GDPPR whose ethnicity data were obtained from GDPPR, from the link to HES-APC, or were not recorded.

#### Inconsistency: multiple records

We study the presence of multiple ethnicity codes for an individual within the GDPPR records and within the HES-APC records, and we compared the prevalence of individuals with multiple codes in GDPPR and HES-APC.

To study it within the GDPPR records, the individual’s SNOMED concepts were converted into the corresponding 19 NHS ethnicity code categories. For individuals who had two co-existing NHS ethnicity codes, the frequency of each co-existing pair was determined. If a patient had more than two co-existing ethnicity codes present, a count of one was added for each pairing.

#### Inconsistency: potential discrepancies between classifications

To study potential discrepancies and misclassifications between the different ethnicity classifications, we studied the mappings between SNOMED and NHS ethnicity codes and between NHS ethnicity codes and high-level ethnicity groups.

#### Granularity: from high-level categories to SNOMED concepts

Here “granularity” refers to the degree of detail, i.e., sub-groups within an ethnicity group. Definitions of the most recent SNOMED record in GDPPR individuals were explored.

Data were prepared using Python V.3.7 and Spark SQL (V.2.4.5) on Databricks Runtime V.6.4 for Machine Learning. Data were analysed using Python in Databricks and RStudio (Professional) Version 1.3.1093.1 driven by R Version 4.0.3.

## Results

### Completeness of ethnicity data

**Fig. 4:**
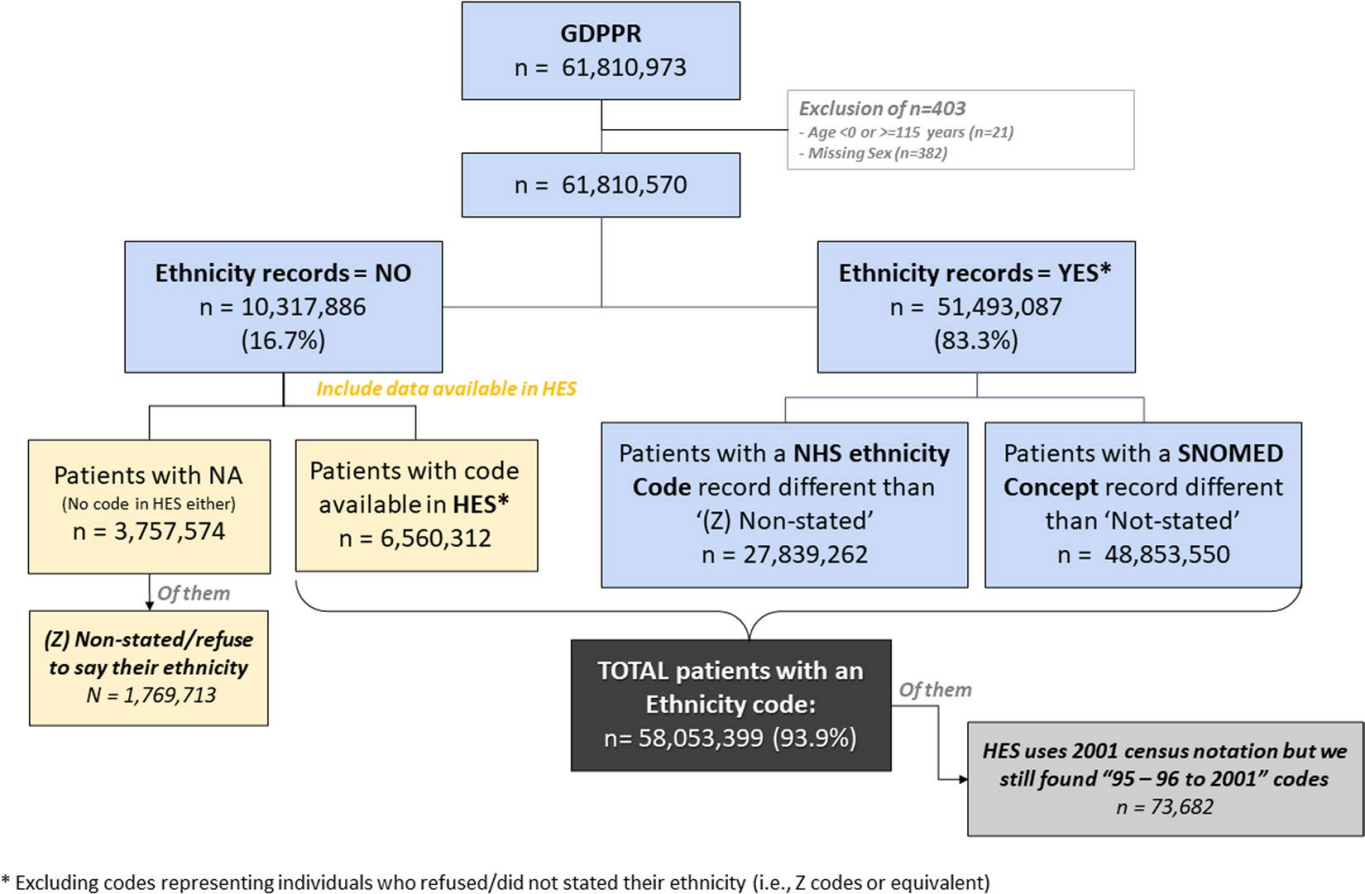
Flow chart of availability of ethnicity records for individuals present in GDPPR. Abbreviations: GDPPR, General Practice Extraction Service (GPES) Data for Pandemic Planning and Research; HES-APC, hospital episode statistics; NHS, National Health Service in the UK; SNOMED, SNOMED-CT records containing ethnicity concepts; NA, not available ethnicity.

We identified 61,810,570 individuals with unique identifiers in the GDPPR dataset on 23^rd^ April 2022. We excluded 403 of these individuals for invalid age or missing sex. Of the remaining, 51,135,903 (83.3%) had an ethnicity code recorded, including those whose ethnicity was recorded but as Unknown, whereas 10,674,667(16.7%) did not have any record for ethnicity. The recorded ethnicity groups included White (77.3%), Asian/Asian British (9.8%), Black/Black British (3.6%), Other Ethnic Groups (3.6%, Mixed (2.2%), and Unknown ethnicity (3.2%) (Figure S1). When linked with HES-APC, the proportion of those without any ethnicity record reduced from 16.7% to 6.1% (Fig. 4).

Individuals with missing ethnicity data were generally younger, with a median age [IQR] of 35·0 [22·0, 53·0] years vs 42·0 [24·0, 61·0] years for those with ethnicity from GDPPR and 36·0 [18·0, 58·0] years for those with ethnicity linked from HES-APC. A greater proportion of those with missing ethnicity were male (58·6%) than those with ethnicity from GDPPR (48·9% male) or HES-APC (54·0% male) (Table 2). They also had fewer comorbidities (Table 3) and a greater proportion came from the South East and South West regions of England (Figure S2). Individuals aged 18–29 years had between 5·7% and 9% more missing ethnicity data than any other age group (Table 2).

**Table 2:** Comparison of individuals with and without an ethnicity record in GDPPR or linked from HES-APC: general characteristics.

Individuals with Characteristics	Ethnicity recorded in GDPPR* (N = 51,493,087)	Ethnicity linked from HES-APC* (N = 6,560,312)	Ethnicity not stated/recorded** (N = 3,757,171)	Total GDPPR (N = 61,810,570)
**Age, years:**				
Mean (SD)	42·6 (23·5)	39·1 (25·1)	38·1 (20·9)	41·9 (23·6)
Median [Min, Max]	42·0 [0, 115]	36·0 [0, 113]	35·0 [0, 115]	41·0 [0, 115]
**Age groups, years:**				
0–17	9345962 (18·2%)	1539936 (23·5%)	634290 (16·9%)	11520188 (18·6%)
18–29	7203161 (14·0%)	1137551 (17·3%)	866021 (23·0%)	9206733 (14·9%)
30–39	7622783 (14·8%)	859347 (13·1%)	645268 (17·2%)	9127398 (14·8%)
40–49	6809996 (13·2%)	707735 (10·8%)	496539 (13·2%)	8014270 (13·0%)
50–59	6966406 (13·5%)	777275 (11·8%)	461384 (12·3%)	8205065 (13·3%)
60–69	5706615 (11·1%)	601874 (9·2%)	330270 (8·8%)	6638759 (10·7%)
70–79	4613388 (9·0%)	468309 (7·1%)	195175 (5·2%)	5276872 (8·5%)
80–89	2462254 (4·8%)	328554 (5·0%)	94656 (2·5%)	2885464 (4·7%)
90+	762522 (1·5%)	139731 (2·1%)	33568 (0·9%)	935821 (1·5%)
**Sex:**				
Female	26311290 (51·1%)	3015261 (46·0%)	1554863 (41·4%)	30881414 (50·0%)
Male	25181797 (48·9%)	3545051 (54·0%)	2202308 (58·6%)	30929156 (50·0%)
**BMI (kg/m2):**				
Mean (SD)	27·3 (6·63)	27·3 (6·80)	26·4 (6·67)	27·2 (6·65)
Median [Min, Max]	26·4 [10·0, 80·0]	26·5 [10·0, 80·0]	25·5 [10·0, 79·9]	26·4 [10·0, 80·0]
Missing	26413905 (51·3%)	4731250 (72·1%)	2905792 (77·3%)	34050947 (55·1%)
**IMD decile:**				
1 (most deprived)	5030900 (9·8%)	611062 (9·3%)	290292 (7·7%)	5932254 (9·6%)
2	5002481 (9·7%)	585319 (8·9%)	310009 (8·3%)	5897809 (9·5%)
3	5026837 (9·8%)	586855 (8·9%)	327026 (8·7%)	5940718 (9·6%)
4	4821880 (9·4%)	630567 (9·6%)	345865 (9·2%)	5798312 (9·4%)
5	4670559 (9·1%)	623723 (9·5%)	331139 (8·8%)	5625421 (9·1%)
6	4657873 (9·0%)	627814 (9·6%)	354218 (9·4%)	5639905 (9·1%)
7	4489880 (8·7%)	620733 (9·5%)	340289 (9·1%)	5450902 (8·8%)
8	4467923 (8·7%)	632435 (9·6%)	338721 (9·0%)	5439079 (8·8%)
9	4365799 (8·5%)	634458 (9·7%)	338340 (9·0%)	5338597 (8·6%)
10 (less deprived)	4292948 (8·3%)	640425 (9·8%)	362403 (9·6%)	5295776 (8·6%)
Missing	4666007 (9·1%)	366921 (5·6%)	418869 (11·1%)	5451797 (8·8%)
Geographic regions***:				
East Midlands	3471792 (6·7%)	336184 (5·1%)	234275 (6·2%)	4042251 (6·5%)
East of England	4462265 (8·7%)	480993 (7·3%)	373491 (9·9%)	5316749 (8·6%)
London	8133607 (15·8%)	743482 (11·3%)	541694 (14·4%)	9418783 (15·2%)
North East	1841607 (3·6%)	211971 (3·2%)	95445 (2·5%)	2149023 (3·5%)
North West	5975649 (11·6%)	1024996 (15·6%)	336139 (8·9%)	7336784 (11·9%)
South East	6678501 (13·0%)	1163911 (17·7%)	645049 (17·2%)	8487461 (13·7%)
South West	3484388 (6·8%)	741784 (11·3%)	336138 (8·9%)	4562310 (7·4%)
West Midlands	4994436 (9·7%)	643818 (9·8%)	235647 (6·3%)	5873901 (9·5%)
Yorkshire and The Humber	4773116 (9·3%)	460537 (7·0%)	319327 (8·5%)	5552980 (9·0%)
Missing	7677726 (14·9%)	752636 (11·5%)	639966 (17·0%)	9070328 (14·7%)
**Death**	995430 (1·9%)	238372 (3·6%)	39434 (1·0%)	1273236 (2·1%)
**Smoke use record**	20509149 (39·8%)	2305227 (35·1%)	1064288 (28·3%)	23878664 (38·6%)
**Heavy alcohol use**	796064 (1·5%)	82195 (1·3%)	29064 (0·8%)	907323 (1·5%)

*Excluding individuals who refused to state their ethnicity (NHS ethnicity code was ‘Z’). **Group composed by individuals who refused to state their ethnicity (NHS ethnicity code was ‘Z’) and those whose ethnicity was not recorded. ***Geographic regions reported in the table belongs to the nine official regions of England. Abbreviations: IMD, index of multiple deprivation; NHS-APC, National Health Service for admitted patient care in the UK; SD, standard deviation.

**Table 3:** Comparison of individuals with and without an ethnicity record in GDPPR or linked from HES-APC: clinical diagnostics.

Individuals with Clinical diagnostics	Ethnicity recorded in GDPPR* (N = 51,493,087)	Ethnicity linked from HES-APC* (N = 6,560,312)	Ethnicity not stated/recorded** (N = 3,757,171)	Total GDPPR N = 61,810,570)
**Atrial fibrillation**	1300604 (2·5%)	173928 (2·7%)	36506 (1·0%)	1511038 (2·4%)
**Acute myocardial infraction**	872067 (1·7%)	99550 (1·5%)	20305 (0·5%)	991922 (1·6%)
**Chronic kidney disease**	2147541 (4·2%)	245624 (3·7%)	59129 (1·6%)	2452294 (4·0%)
**COPD**	1123202 (2·2%)	104455 (1·6%)	24733 (0·7%)	1252390 (2·0%)
**Heart failure**	701978 (1·4%)	88202 (1·3%)	16738 (0·4%)	806918 (1·3%)
**Pulmonary embolism**	5257 (0·0%)	529 (0·0%)	129 (0·0%)	5915 (0·0%)
**Cancer**	6203838 (12·0%)	604350 (9·2%)	208500 (5·5%)	7016688 (11·4%)
**Dementia**	501322 (1·0%)	76545 (1·2%)	16203 (0·4%)	594070 (1·0%)
**Diabetes**	3703702 (7·2%)	321356 (4·9%)	106695 (2·8%)	4131753 (6·7%)
**Hypertension**	7860172 (15·3%)	809692 (12·3%)	252239 (6·7%)	8922103 (14·4%)
**Liver disease**	174646 (0·3%)	17650 (0·3%)	4649 (0·1%)	196945 (0·3%)
**Obesity**	2611818 (5·1%)	241761 (3·7%)	82667 (2·2%)	2936246 (4·8%)
**Stroke**	1048990 (2·0%)	131630 (2·0%)	27982 (0·7%)	1208602 (2·0%)

*Excluding individuals who refused to state their ethnicity (NHS ethnicity code was ‘Z’). **Group composed by individuals who refused to state their ethnicity (NHS ethnicity code was ‘Z’) and those whose ethnicity was not recorded. ***Geographic regions reported in the table belongs to the nine official regions of England. Abbreviations: COPD, chronic obstructive pulmonary disease; NHS-APC, National Health Service for admitted patient care in the UK.

### Assessment of multiple ethnicity records

About 1·4% of individuals with an original NHS ethnicity code record and 16·0% of individuals with a converted SNOMED concept record had multiple different ethnicity codes (Table S3). Excluding the *Not stated* (Z) code reduced inconsistencies (to 1·2% and 10·3%, respectively). In contrast, 38·0% of individuals in GDPPR with at least one ethnicity record in HES-APC (n = 46,804,958) had multiple inconsistent records, dropping to 19·0% when the *Not stated* (Z) code was excluded.

Ethnicity codes most frequently found in individuals with more than one reported code in GDPPR were *British* (A), *Any other White background* (C), *Not stated (Z)*, *Any other ethnic group* (S), and *Any other Asian background* (L) (Figure S3). The most common ethnicity code combinations were *British* (A) – *Any other White background* (C), *British* (A) – *Not stated* (Z), and *Any other White background* (C) – *Any other ethnic group* (S). When White ethnicity codes (A, B, and C) were excluded, the most common pairs of minority ethnicity codes were *Any other Asian background* (L) – *Any other ethnic group* (S), *African* (N) – *Any other Black background* (P), and Indian (H) *– Any other Asian background* (L) (Figure S4).

### Granularity of ethnicity data

Figure S2 maps the distinct levels of ethnicity concepts from the different data sources to one another. SNOMED currently gives the most granular ethnicity records, with 489 SNOMED concepts representing ethnicity within the NHS England SDE (Table S1). However, only 255 (52·1%) of these codes were used at least once in the extracted individuals’ records. The remaining 234 (47·8%) codes were not assigned to any individual. Figure S5 shows the five most frequently used SNOMED concepts mapped to each NHS ethnicity code. Table S4 displays the number of individuals per SNOMED concept in GDPPR.

### Diversity in SNOMED concepts

SNOMED concepts were substantially diverse. Of the 255 codes in use, 162 (63·5%) contained an ethnicity/race concept, 5 (2·0%) included a religion, 187 (73·3%) referenced a geographical region, and 60 (23·5%) referenced a language. Full list is available at Table S5.

### Potential discrepancies and misclassifications

Some inconsistency was found in the aggregation of NHS ethnicity categories into the high-level ethnicity groups. According to the 2011 and 2021 England and Wales census classifications, *Gypsy/Irish Traveller* (T) falls within the higher-level category *White*, and *Chinese* (R) within *Asian*. In contrast, the NHS ethnicity classification included *Gypsy/Irish Traveller* (T) and *Chinese* (R) within the higher-level category *Other Ethnic Groups*, following the ONS Census 2001 classification (see classification algorithm used in the CVD-COVID-UK/COVID-IMPACT Consortium in Table S2).

**Fig. 5:**
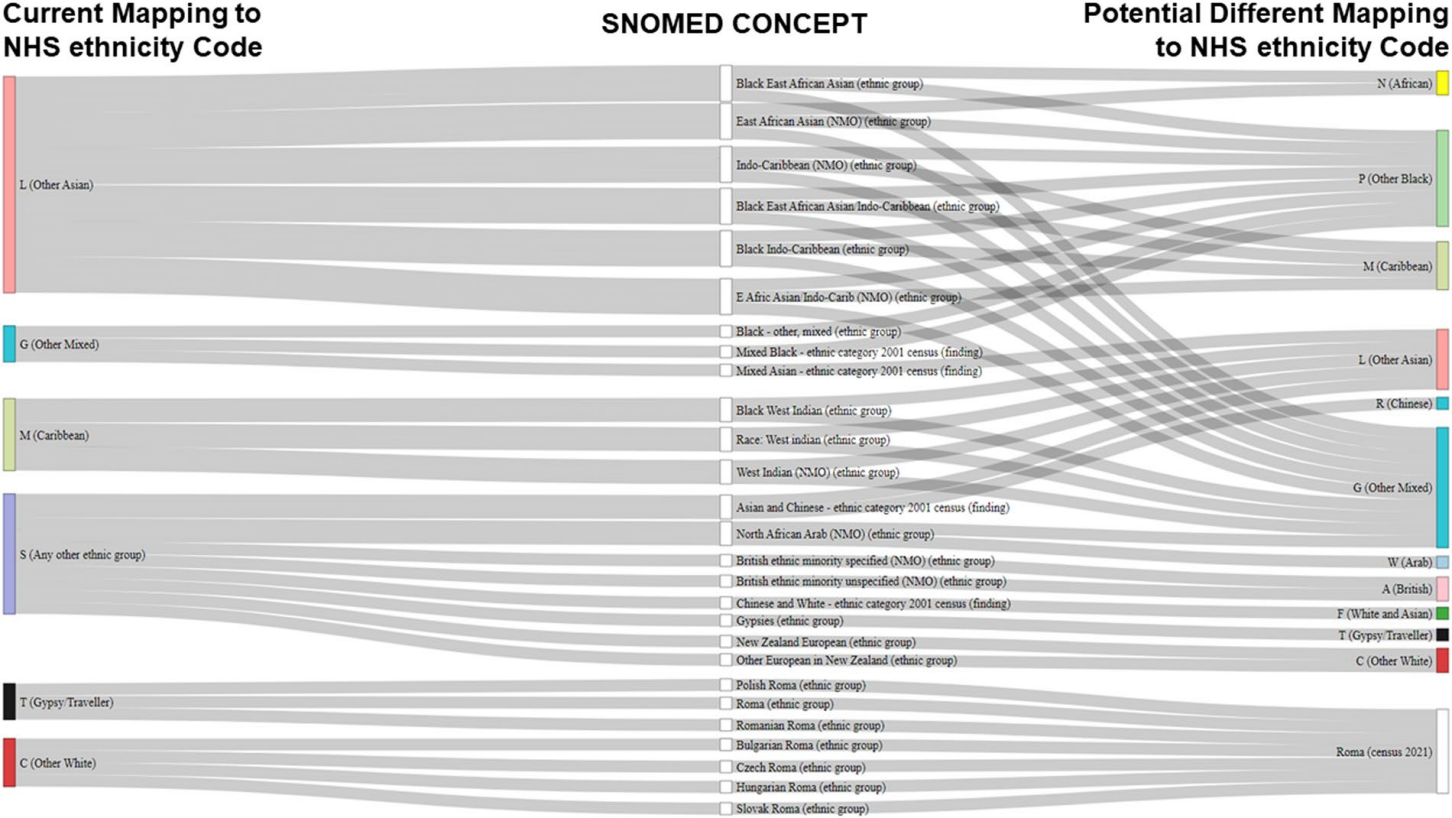
Sankey plot showing potential discrepancies between SNOMED concepts and NHS ethnicity codes mapping. Abbreviations: NHS, National Health Service in the UK; SNOMED, SNOMED-CT records containing ethnicity concepts.

Further discrepancies were found in the grouping of SNOMED concepts into NHS ethnicity categories. A mapping algorithm could not be traced. Given the lack of documentation on the mapping of SNOMED concepts to NHS ethnicity code, several potential discrepancies were observed which should be carefully (re)considered by researchers in future (Fig. 5): for instance, concepts including a variant of “Black East African Asian/Indo-Caribbean” were assigned to *Any other Asian background* (L). However, there is no clarification as to whether these concepts are mapped more accurately there than others, such as *Other ethnic group* or *Black/African/Caribbean/Black British*. Likewise, variants of “Black West Indian” were mapped to *Caribbean* (M), although a proportion of these individuals may have Asian legacy. Arguably, the three ‘Mixed’ concepts within *Any other background* (G) may be better grouped in more specific categories, such as “Black - other, mixed” within *Black background* (P). Several concepts contained by *Any other ethnic group* (S) could also be placed in more specific categories. For example, the 2001 census category “Asian and Chinese” is linked to *Any other ethnic group* (S) instead of *Chinese* (R) or *Any other Asian background* (L). Similarly, some SNOMED concepts include the concept “Roma”. As the ONS 2021 census included the new category *White: Roma*, the mapping could be updated to reflect this change.

### Facilitating data reuse in future research

The curation process has organised ethnicity data into a hierarchical mapping. This allows the data source to be reused by future researchers using ethnicity information. The publicly available R code can be used to extract the most up-to-date ethnicity records for future research. This allows the necessary flexibility for using the data in observational research such as retrospective cohort studies, as well as potentially help with clinical trial selection such as NHS DigiTrials service.

## Discussion

Errors in health care can impact patient care and outcomes as well as increase costs to the care system^25^ and affect public trust^26^. Biased ethnicity knowledge could potentially lead to biased healthcare decision-making and to patients receiving inappropriate or no care. Correct identification of ethnicity is an essential first step to understanding inequities between ethnicities. Despite its complexity, researchers should aim to include ethnicity in their analyses. The results presented here can be used to further the use of ethnicity in future research.

Among those whose ethnicity was recorded, the proportion of individuals with White Black/Black British and Mixed ethnicity were −3.7%, −0.6% and −0.7% lower, respectively, whilst Asian/Asian British and Other Ethnic groups were and 0.2% and 1.4% higher, as compared to 2021 census estimates^27^. Therefore we consider GDPPR as a representative data source for the England population.

### Completeness of ethnicity records

We found that over 83·3% of individuals in England’s primary care system had at least one ethnicity recorded; increasing to 93·9% when linked to hospitalisation records. This result represents a greater level of completeness than reported in other routinely collected GP records^28^, and highlights the usefulness of linking data across primary and secondary care to maximise ethnicity data completeness. Individuals with missing ethnicity were younger, more likely to be male and living in the southern regions of England, and had fewer comorbidities than individuals with recorded ethnicity. It may be speculated that this group may be representative of generally healthy individuals or those otherwise not inclined to seek healthcare. In other words, most of individuals with Unknown ethnicity might not be using the health care system very often, which decreases the probability to record data. Similar results have been reported in other UK data sources. For instance, Mathur *et al*. observed higher rates of ethnicity records for individuals aged 40 to 79 years in the Clinical Practice Research Datalink (CPRD) and HES data sources than older or younger individuals^8^. Petersen at al. found that, among people aged 18–65 years, men were less likely to have health indicators recorded than women in the Health Improvement Network (THIN)^29^.

### Granularity of ethnicity data

Most studies collapse the available ethnicity concepts into five (e.g., Asian, Black/African/Caribbean, White, Mixed, Other Ethnic Groups) or six (the aforementioned and Unknown ethnicity) categories^11^. However, some studies have accounted for greater distinctions by exploring ethnic minorities such as Bangladeshi in the UK and Hispanic/Latinos in the US^30^. This work describes for the first time more than 250 patient-identified ethnicity sub-groups in England.

Ethnicity data in healthcare and national statistics are captured for different purposes, partly explaining some of the differences among NHS and census ethnicity categories. For instance, GDPPR is directly intended for patient care; HES has a more administrative nature linked to payments; while the ONS collects information from the UK population, including ethnicity, in a census held approximately every 10 years, most recently in 2021^7,13^. To allow for the emergence of new ethnicity groups, the census questionnaire allows free-text answers^13^. After pooling all information, the ONS reports the groups that, in their understanding, best represent the existing diversity in the UK. Updated ethnicity groups are then shared with the NHS, which uses this information to update the ethnicity categories used in their data sources.

The 2011 Census published by the ONS was the gold standard for ethnicity recording in England and Wales until the recent publication of the 2021 Census^31^. However, not all NHS sources base their categories on the same census. For example, HES-APC uses 2001 Census categories, whereas GDPPR uses 2011 census categories. This discrepancy creates uncertainties and data mismatching between different datasets. For example, HES-APC does not include the ethnicity categories *Arab* (W) and *Gypsy/Irish Traveller* (T)^32^. This highlights once more the importance of linking data across primary and secondary care, in this case, to maximise ethnicity data granularity.

Despite differences, we can compare the prevalence of SNOMED concepts used in GDPPR to the 2019 population estimates in England and Wales (2019)^33^. Fewer individuals self-identified as White British (66·8% in GDPPR vs 78·4% in 2019 estimates), Gypsy/Traveller (0·1% vs· 0·03%), or Arab (0·2% vs 0·4%). Higher proportions of individuals self-identified as Chinese (1·2% vs 0·6%), Indian (3·7% vs· 2·8%), Pakistan (2·9% vs 2·3%), or Any other mixed background (0·8% vs 0·5%). And similar percentages self-identified as Bangladeshi (1·1% vs 1·0%), Caribbean (0·9% vs 1·0%), African (2·4% vs 2·3%), White and Asian (0·5% both), White and Black African (0·4% vs 0·3%), or White and Black Caribbean (0·5% both). The 2019 estimates did not include people who had died or individuals with an unknown ethnicity, which might account for these differences.

### Multiple records and potential discrepancies

Our analysis of multiple ethnicity records within GDPPR and HES-APC sources showed relatively similar discrepancy rates when the code for “do not know/refusal” (i.e., *Not stated* (Z)) was excluded (12% and 19%, respectively). However, GDPPR ethnicity data should be prioritised to ensure inclusion of *Arab* (W) and *Gypsy/Irish Traveller* (T) ethnicities, as well as to reduce the inclusion of older notations such as the “Codes from 1995–1996 to 2000–2001”. Using the prioritisation algorithm from our analysis, the impact of this legacy classifications represented less than the 0.13% of individuals with an ethnicity record and less than 0.12% of all individuals registered in GDPPR. Nonetheless, we considered that using an old classification system is preferred, rather than registering it as missing ethnicity, but researchers may decide differently on a project basis.

Within patient records, the most frequently coexisting codes would be placed in the same higher-order group. For example, codes for *African* (N) and *Any other Black background* (P) often appear for the same individual and would both be grouped within the high-level category Black/African/Caribbean/Black British. The use of higher-level groupings can therefore resolve some conflicting cases by reducing granularity. However, it cannot resolve conflicts where different Mixed categories coexist in the same record, such as *Any Other Mixed Background* (G) occurring alongside *British* (A), *Any Other White Background* (C), or *African* (N). Higher-level Mixed groupings may therefore include more ambiguous ethnicity concepts. The *British* (A) code had frequent conflicting pairings with *Indian* (H), *Any other Asian background* (L), and *Caribbean* (M), suggesting inconsistencies in individuals’ perceptions of their nationality and ethnicity when self-reporting ethnicity.

The grouping algorithm used can also be a source of inconsistencies. For instance, including *Chinese* (R) and *Gypsy/Irish Traveller* (T) within Other Ethnic Groups instead of the established high-level ethnicity groups might be preferred for certain studies, but should not be by default.

Uncertainty regarding mapping of international SNOMED-CT ethnicity concepts to NHS ethnicity codes highlighted the need for better documentation of underlying processes. SNOMED concepts available in GDPPR data account for different, more granular ethnicity groups than NHS ethnicity codes, enabling greater diversity in ethnicity groups to be represented. The descriptions of the observed SNOMED concepts included ethnicity, race, religion, and geographic location, among others. However, many concepts require some aggregation due to their limited use within very large datasets, such as the one explored here. Most research based on NHS data uses wider categories, rather than the highly specific concepts captured by the SNOMED concepts. The large variety and complexity of ethnicity codes can make collapsing and comparing codes difficult, regardless of whether NHS ethnicity codes or high-level ethnicity groups are used. Although using these more general groupings allows researchers to achieve a minimum sample size while protecting individual identities, the cost is the uncertainty of how accurate these bigger groups are. In addition to the improvement of mapping quality, having better documentation of how the observed SNOMED concepts were defined could help in the comparison of these groups with classifications from other countries.

### Strengths and limitations of this study

To our knowledge, this work is the first attempt to curate and describe the full breadth and depth of patient self-identified ethnicity using more than 250 ethnicities among over 61 million individuals in England.

GDPPR is a collection of de-identified person-level primary care data (linked to secondary and tertiary care) for one of the world’s largest research-ready population-wide electronic health records databases and housed within a trusted research environment, NHS England’s SDE service for England. This extensive observational dataset, with its large number of ethnicity groups and sub-groups, has the potential to deepen our understanding of ethnicity in health data and its potential to improve real-world evidence generation.

Despite the exclusion of individuals who died before 1^st^ November 2019, GDPPR can provide a reliable picture of the existing ethnic diversity for studies including individuals registered in the England primary care system after November 2019, like ours. We considered GDPPR as a representative data source for the England population diversity when compared to the UK 2021 census^27^. The slight variations that may be observed, with 1.4% higher representation of Other Ethnic groups being the largest difference, may be explained by our decision not to restrict our analysis to only alive individuals like most researchers would use in their research (in other words, we include all individuals registered in GDPPR with valid inclusion criteria, including those individuals who died between November 2019 and April 2022). However, studies aiming to analyse the diversity of the population before this date may be biased and, therefore, not be representative.

This study provides a first, detailed curation of ethnicity data for re/use in research. The observed findings are highly representative of the England population: in England, there was a total of 6,700 GP practices containing a total number of 60,389,925 unique NHS identifier from patients who were not deceased by 24 August 2020^34^. Of them, 6,535 GP practices containing 56,441,600 unique identifiers were included within GDPPR. Nevertheless, we do not disregard the possibility that patients registered at multiple practices with different identifiers could been counted more than once. However, the impact of this is reduced by the Master Person Service algorithm, which increases the quality of the data by matching and linking person-records within and across the different NHS sources^17^ In other words, the algorithm links the different NHS identifiers from the same single patient not only within GDPPR but also across other linked datasets such as HES-APC tables, and assigns a unique anonymised identifier (named *Person_ID* within the NHS England SDE) that is later used by the SDE user. Further studies are required to assess its accuracy in GDPPR records.

Ethnic diversity is better captured in SNOMED concepts than other existing classifications. Future observational research with study-specific sample sizes may need to consider combining smaller ethnicity categories into larger groups for study feasibility. The detailed representation of the England population described here also means the observed ethnicity groups may not be equivalent or transportable to other countries. Additionally, there is no perfect solution for conflicting codes in the same individual, especially for codes that cannot be reconciled (e.g., White, Black). Of the different available approaches, we used the most recent SNOMED concepts in an individual’s record when exploring granularity in GDPPR. This approach may have affected the prevalence of very small minority groups, including the 234 codes that were not linked to any user. An alternative approach would have been to select the most frequently recorded ethnicity category, which could reduce any potential human error when entering the data into the electronic health system. However, using the most recent codes has the advantage to include any new ethnicity definitions within SNOMED, allowing us to observe a more up-to-date representation of the self-perception of ethnicity of the population.

Whilst improvements to data collection at source would be welcome, much more can be done with currently available ethnicity data than is typically seen in the literature. This is important to be done now more than ever, as routinely collected ethnicity data are increasingly used in the era of real-world analytics and large-scale trials. For instance, improvements required in ethnicity mapping between classifications were identified in this paper. Additionally, this study demonstrates the importance of linking data across primary and secondary care to maximise the ascertainment, completeness, and granularity of ethnicity data, and the application of better ethnicity coding in big health data.

The details of using ethnicity provided in this paper may not only help researchers to improve the representation of the population diversity in their research, but can also be used to conduct much more personalised medicine such as tailoring prognostic models to the 19 ethnicity groups. Accurate ethnicity data will lead to a better understanding of individual diversity, which will help to address disparities and influence policy recommendations that can translate into better, fairer health for all. This, in turn, shows that the effort of collecting ethnicity and using it in research is more than worthwhile.

## Ethical approval

The North East – Newcastle and North Tyneside 2 research ethics committee provided ethical approval for the CVD-COVID-UK/COVID-IMPACT research programme (REC no: 20/NE/0161) to access, within secure trusted research environments, unconsented, whole-population, de-identified data from electronic health records collected as part of patients’ routine healthcare. Our project (proposal CCU037, short title: Minimising bias in ethnicity data) agreed to the objectives of the consortium’s ethical and regulatory approvals and was authorised by the BHF Data Science Centre’s Approvals and Oversight Board. Approved researchers (MPM, AD, SK) conducted the analyses within the NHS England’s SDE via secure remote access. Ensuring the anonymity of individuals, only summarised-aggregated results that were manually reviewed by the NHS England ‘safe outputs’ escrow service were exported from the SDE.

## Patient and public involvement

A panel of four PPI members were recruited to work on the study team and oversee the project. In addition, a wider stakeholder group representing a wide range of different ethnicities was recruited for three online meetings to get input into the study design, review initial results and finally to consider how to disseminate these results to the public. This group led on the design of a poster and infographic to share the results with the public and encourage them to “Be proud of your ethnicity”.

### Supplementary information

Supplementary Information

Supplementary Information - Supplementary Tables on excel

## Data Availability

The data used in this study are available in NHS England’s Secure Data Environment (SDE) service for England (https://digital.nhs.uk/services/secure-data-environment-service). The CVD-COVID-UK/COVID-IMPACT programme led by the BHF Data Science Centre (https://bhfdatasciencecentre.org/) received approval to access data in NHS England’s SDE service for England from the Independent Group Advising on the Release of Data (IGARD) (https://digital.nhs.uk/about-nhs-digital/corporate-information-and-documents/independent-group-advising-on-the-release-of-data) via an application made in the Data Access Request Service (DARS) Online system (ref. DARS-NIC-381078-Y9C5K) (https://digital.nhs.uk/services/data-access-request-service-dars/dars-products-and-services). The CVD-COVID-UK/COVID-IMPACT Approvals & Oversight Board (https://bhfdatasciencecentre.org/areas/cvd-covid-uk-covid-impact/) subsequently granted approval to this project to access the data within NHS England’s SDE service for England. The de-identified data used in this study were made available to accredited researchers. Those wishing to gain access to the data should contact bhfdsc@hdruk.ac.uk in the first instance.

## Code availability

All code for data preparation and analysis are publicly available on GitHub (https://github.com/BHFDSC/CCU037_01).
