# Supplementary material for: Ethnicity data resource in population-wide health records: completeness, coverage and granularity of diversity
Source: Sci Data. 2024 Feb 22;11:221. doi: 10.1038/s41597-024-02958-1 (PMC10883937; doi:10.1038/s41597-024-02958-1)
Supplement: Supplementary file 1 — Supplementary Information [file 41597_2024_2958_MOESM1_ESM.docx]

**Supplementary Figures**

### Figure S1. Representation of the hierarchical structure from “high-level ethnic groups” to NHS ethnicity Codes and the observed 255 SNOMED concepts.

The “observed SNOMED concepts” refers to the 255 of 489 concepts available in the SDE that were used at least once in the GDPPR dataset (n = 51,135,903). Branches from the same high-level ethnic groups are coloured using the same colour or shade. Abbreviations: High-level ethnic groups, general ethnicity classification groups from the Office for National Statistics commonly used in research; NHS, National Health Service in the UK; SNOMED, SNOMED-CT records containing ethnicity concepts; SDE, Secure Data Environment for the NHS England.


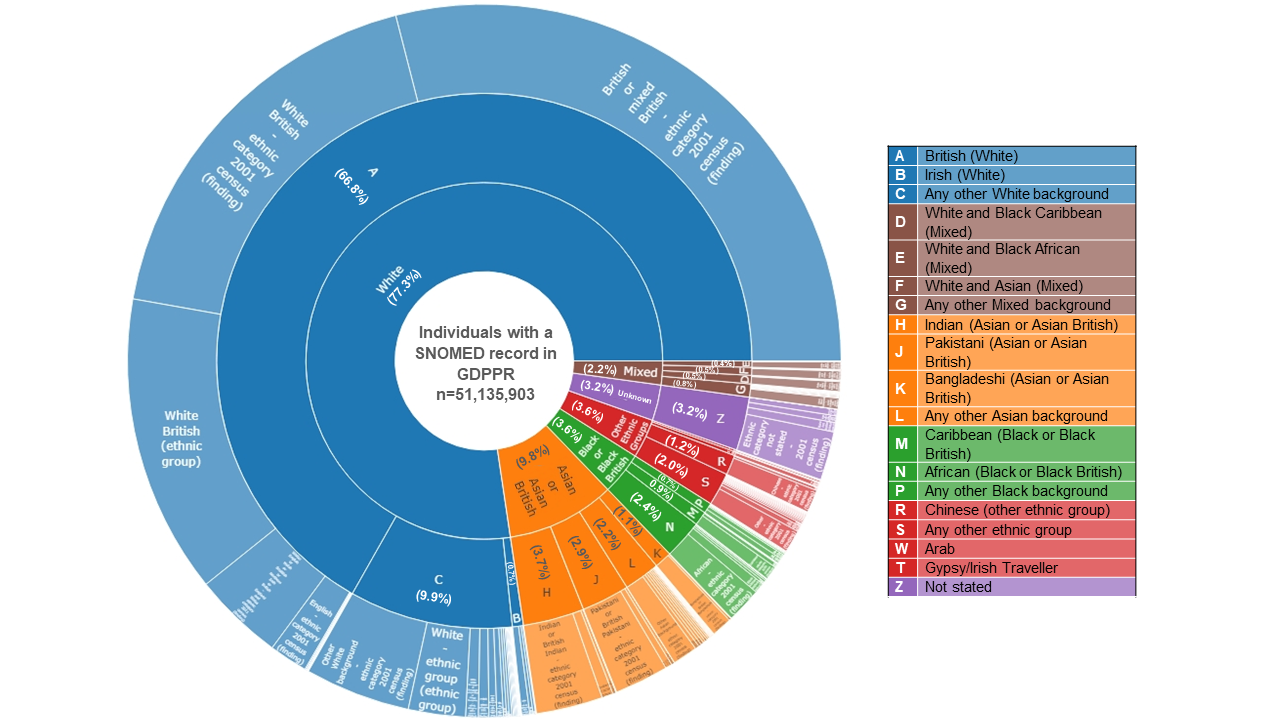


### Figure S2. England regions with percentage of individuals with no ethnicity records.

In GDPPR, 14.7 % did not have a post code record and could not be allocated in the map.
The shown values are the frequency of “individuals with no ethnicity divided by the total individuals” in each of the England regions. Abbreviations: GDPPR, General Practice Extraction Service (GPES) Data for Pandemic Planning and Research.


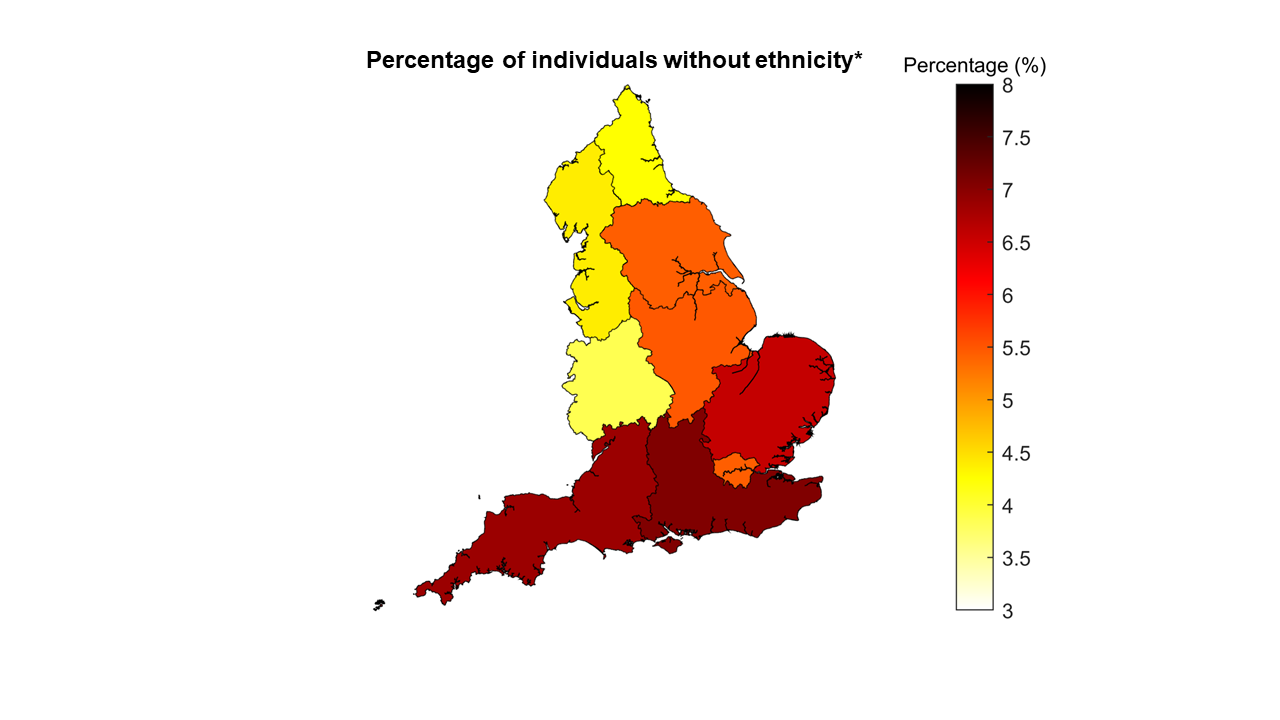


### Figure S3. Ethnicity codes that were most frequently found in records with more than one reported code


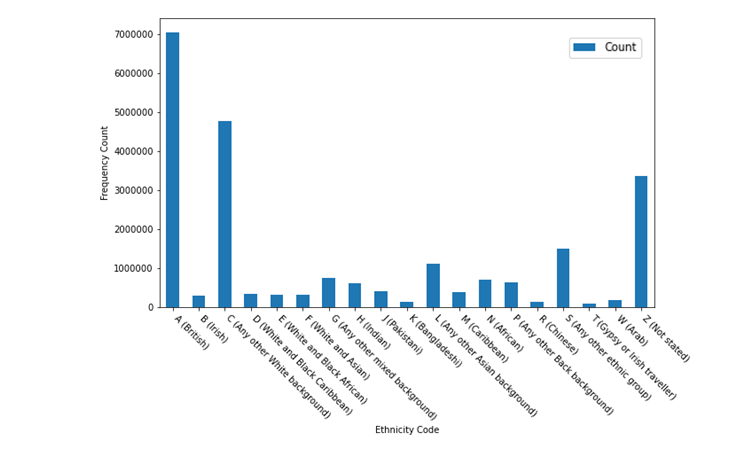


### Figure S4. Frequency of pairwise occurrence when a) including all ethnicity codes and b) excluding the majority population with White ethnicity codes (A, B or C).

NHS ethnicity codes: A, British; B, Irish; C, Any other White background, D, White and Black Caribbean; E, White and Black African; F, White and Asian; G, Any other mixed background; H, Indian; J, Pakistani; K, Bangladeshi; L, Any other Asian background; M, Caribbean; N, African; P, Any other Black background; R, Chinese; S, Any other ethnic group; T, Traveller; W, Arab; Z, Not stated. Abbreviations: NHS, National Health Service in the UK.

a)


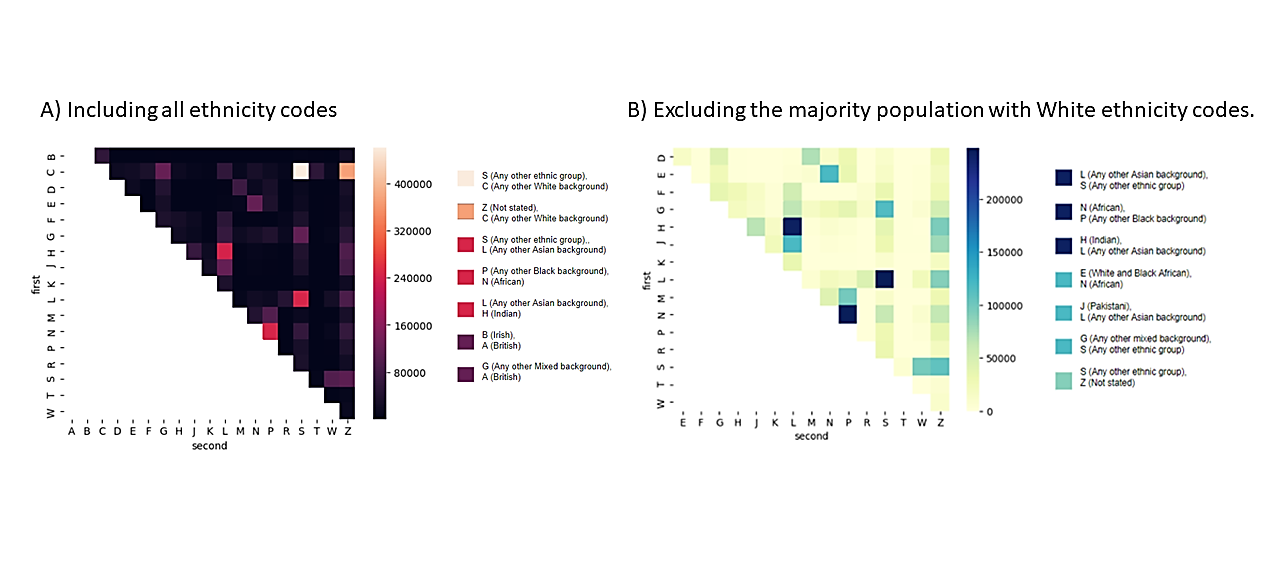


b)

### Figure S5. Top five SNOMED concepts most frequently used in each NHS ethnicity Code level, arranged by “high-level ethnic groups”: a) Asian/Asian British, b) Black/African/Caribbean/Black British, c) Mixed, d) Other Ethnic Groups, e) White and f) Unknown.

NHS ethnicity codes: A, British; B, Irish; C, Any other White background, D, White and Black Caribbean; E, White and Black African; F, White and Asian; G, Any other mixed background; H, Indian; J, Pakistani; K, Bangladeshi; L, Any other Asian background; M, Caribbean; N, African; P, Any other Black background; R, Chinese; S, Any other ethnic group; T, Traveller; W, Arab; Z, Not stated. Abbreviations: High-level ethnic groups, general ethnicity classification groups from the Office for National Statistics commonly used in research; NHS, National Health Service in the UK; SNOMED, SNOMED-CT records containing ethnicity concepts.


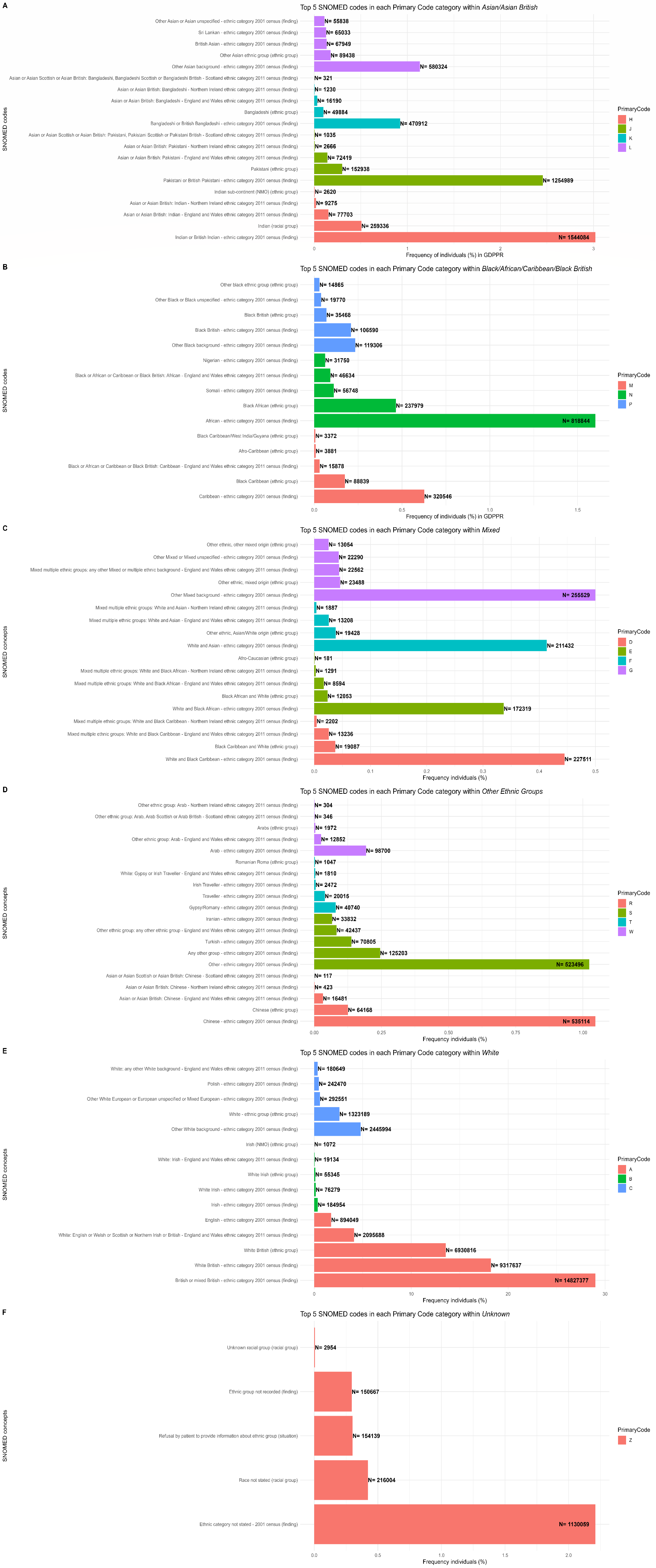

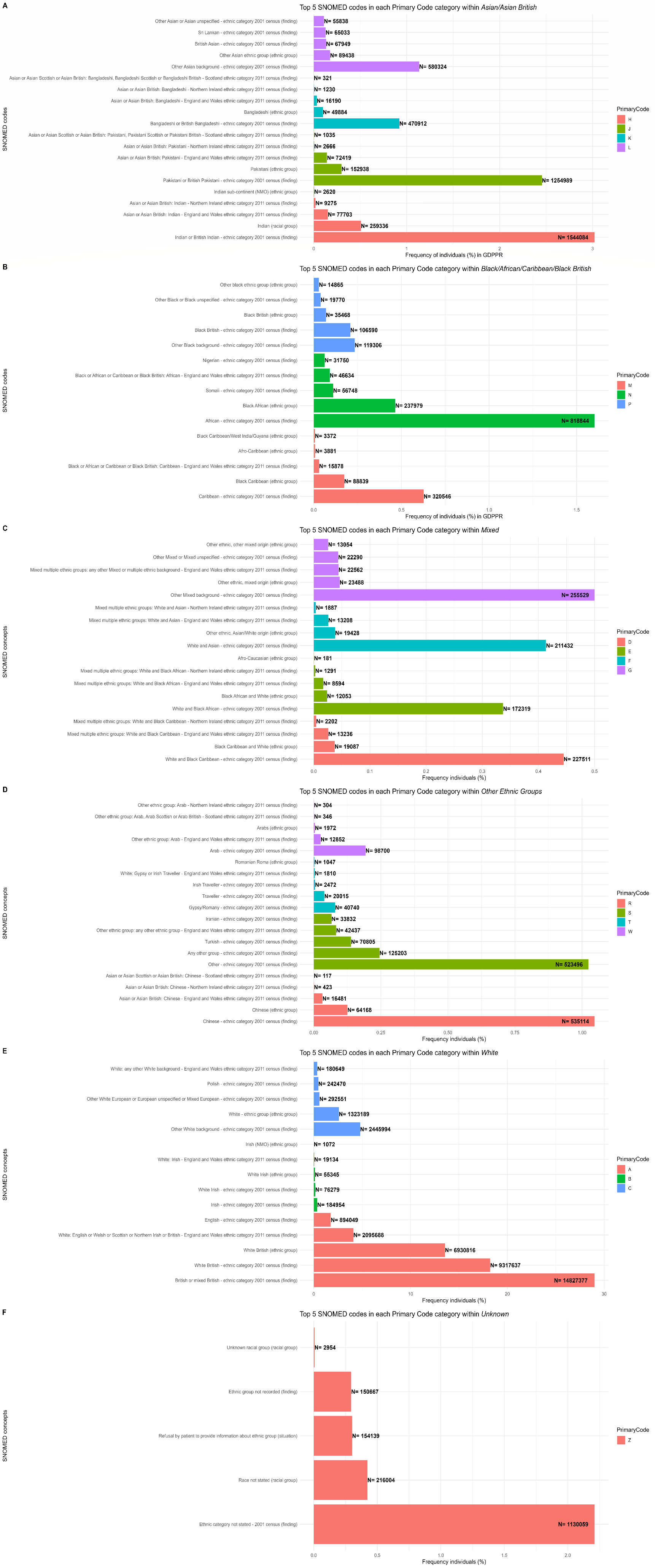


c)

b)

a)

e)

f)

d)

**Supplementary Tables**

### Table S1. Mapping of SNOMED-CT concepts containing ethnicity within the NHS ethnicity codes.

Size of the table too large, see *Supplementary_Tables_on_excel.xlsx* to obtain the full table. This table contains the mapping used for composing Fig 4, and Supplementary Fig 4 and 5.

### Table S2. Algorithm to collapse NHS ethnicity codes into the six high-level ethnic categories*:

| CASE WHEN ETHNICITY_CODE IN ('1','2','3','N','M','P') THEN "Black or Black British"             WHEN ETHNICITY_CODE IN ('0','A','B','C') THEN "White"             WHEN ETHNICITY_CODE IN ('4','5','6','L','K','J','H') THEN "Asian or Asian British"             WHEN ETHNICITY_CODE IN ('7','8','W','T','S','R') THEN "Other Ethnic Groups"             WHEN ETHNICITY_CODE IN ('D','E','F','G') THEN "Mixed"             WHEN ETHNICITY_CODE IN ('9','Z','X') THEN "Unknown"             ELSE 'Unknown' END as ETHNIC_GROUP |
| --- |
| *This is the regularly used algorithm within the CVD-COVID-UK/Covid impact consortium. Codes for *Gypsy/Irish Traveller* ('T') and Chinese ('R') should be placed within "White" and "Asian or Asian British", respectively, according to the 2011 and 2021 ONS Census. |

### Table S3. Discrepancies in multiple ethnicity records across GDPPR and HES-APC tables using individuals identified in GDPPR.

| Ethnicity data source and code type used | Individuals with recorded ethnicity*  (n) | Individuals with inconsistent multiple records  (n, %) | | Individuals with inconsistent multiple records, excluding "Z - Not stated" code (n, %) | |
| --- | --- | --- | --- | --- | --- |
| All GDPPR Codes | 53,266,674 | 9,379,041 | 17·0% | 6,453,095 | 12·0% |
| *GDPPR NHS ethnicity Code* | *29,721,460* | *426,194* | *1·4%* | *353,828* | *1·2%* |
| *GDPPR SNOMED concept* | *51,135,903* | *8,163,018* | *16·0%* | *5,260,197* | *10·3%* |
| Linked-HES-APC NHS ethnicity Code | 46,804,958 | 17,704,692 | 38·0% | 8,658,447 | 19·0% |
| **Including* NHS ethnicity *code ‘Z – not stated’ and the SNOMED concepts mapped to it.  Abbreviations: GDPPR, General Practice Extraction Service (GPES) Data for Pandemic Planning and Research; NHS, National Health Service in the UK; HES-APC, hospital episode statistics for admitted patient care; SNOMED, SNOMED-CT records containing ethnicity concepts.* | | | | | |

### Table S4. Number of individuals in GDPPR per SNOMED-CT concept containing ethnicity. Size of the table too large, see *Supplementary_Tables_on_excel.xlsx* to obtain the full table. This table contains the values used to plot Supplementary Fig 4 and 5.

### Table S5. Diversity of SNOMED-CT codes containing ethnicity. SNOMED-CT concepts contains references to race/ethnicity, religion, country/location, language, other such not stated, settled/traveller, census, etc: 1 refers as included in the prior mentioned terms and 0 not included. Last row include the sum of the concepts within the same diverse terms.

Due to large size of the table, only the last row including the sum of the concepts has been included below. See *Supplementary_Tables_on_excel.xlsx* to obtain the full table.

| SNOMED concept Id description  (n=255) | NHS ethnicity code mapped | Race/ethnicity combined | Religion | Country/ location | Other | Language | Settled/ traveller | Census: 0, 2001 or 2011 | NMO | Finding, ethnic group, racial group or situation |
| --- | --- | --- | --- | --- | --- | --- | --- | --- | --- | --- |
| … | … | … | … | … | … | … | … | … | … | … |
| SNOMED CODES (%)  (Total=255) | - | 162  (63.5%) | 5  (2.0%) | 187  (73.3%) | 7  (2.7%) | 60  (23.5%) | 14  (5.5%) | 139  (54.5%) | 24  (9.4%) | Finding: 144 (56.5%)  Ethnic group: 105 (41.2%)  Racial group: 5 (2.0%)  Situation (i.e., refusal): 1 (0.4%) |

**^…^ =** See *Supplementary_Tables_on_excel.xlsx* to obtain full table.
